# Clinical analysis of 25 cases of infant umbilical granuloma treated with topical application of common salt granules

**DOI:** 10.3389/fped.2025.1592198

**Published:** 2025-07-31

**Authors:** Jingfeng Bao, Hailin Li, Donghai Yu, Ling Jiang, Yaqiang Hong

**Affiliations:** Department of Pediatric Surgery, Affiliated Changzhou Children’s Hospital of Nantong University, Changzhou, Jiangsu, China

**Keywords:** common salt, infant, umbilical granuloma, clinical analysis, topical application

## Abstract

**Objective:**

To summarize our experience with topical application of common salt granules for the treatment of infant umbilical granuloma and to evaluate its clinical value.

**Methods:**

A retrospective analysis was conducted on 25 cases of umbilical granuloma treated with common salt granules from January 2024 to June 2024.

**Results:**

All 25 cases were successfully treated, with a mean treatment duration of 2.5 days (range: 1.5–9 days). The cure rate was 100%, with no recurrence or complications observed during a 3-month follow-up.

**Conclusion:**

Topical application of common salt granules appears to be a simple, effective, safe, and cost-effective treatment for infant umbilical granuloma, based on our retrospective analysis. Further studies are needed to confirm these findings.

## 1 Introduction

Umbilical granuloma is the most prevalent umbilical condition in neonates and young children, characterized by a small protrusion of granulation tissue at the base of the umbilicus. It typically develops within 1–2 weeks postpartum, coinciding with the closure of the umbilical cord's fibromuscular ring and the detachment of the umbilical stump. Incomplete epithelialization of the ring can result in excessive granulation tissue growth, leading to the formation of umbilical granuloma (1, 2). If left untreated, the granuloma may become infected, potentially progressing to severe complications such as omphalitis or necrotizing fasciitis (3, 4). Spontaneous resolution of untreated granuloma is unlikely. While there remains no consensus on the optimal treatment approach, topical silver nitrate application is widely adopted as the first-line therapy in most medical institutions. Alternative invasive treatments include surgical electrocautery, cryotherapy, ligation, and excision; however, these methods, though effective, are associated with specific complications and drawbacks (5–9). Recently, the topical application of common salt granules has emerged as a safe and effective treatment option, garnering increasing attention. For instance, a systematic review by Haftu et al. concluded that salt treatment for umbilical granuloma is effective, cheap, available, and easy to apply, with no reported side effects or recurrence (10). Another study by Banerjee et al. reported a 93.91% success rate with common salt application, without any complications or recurrences. These findings suggest that common salt granules could be a viable alternative to traditional treatments. Our institution has successfully treated umbilical granuloma using this method, achieving satisfactory outcomes without any adverse effects. The following details our experience (11). At our institution, we have treated umbilical granuloma using this method and observed positive outcomes with no adverse effects noted.

## 2 Materials and methods

### 2.1 Study design

This single-center retrospective study was conducted to evaluate the clinical efficacy of topical salt granule application in the treatment of umbilical granuloma in infants. All cases included in this study were treated with topical salt granules at our department between January 2024 and June 2024, with a follow-up period of at least three months. The study received approval from the Ethics Committee of Changzhou Children's Hospital, and informed consent was obtained from all parents. Ethical review registered number: 2025-011.

### 2.2 Object of study

This study encompassed 25 neonates who were clinically diagnosed with umbilical granuloma. The diagnoses were confirmed via comprehensive medical history reviews, thorough physical examinations, and imaging studies, ensuring the exclusion of yolk duct remnant malformations and urachal remnant malformations.

#### 2.2.1 Inclusion criteria

(1) The patient's age ranges from 2 to 20 weeks, with a diagnosis of umbilical granuloma. (2) The diagnosis was established based on a comprehensive medical history review, physical examination, and imaging studies, which ruled out other differential diagnosis. (3) There is no prior successful treatment history. For the 3 cases that did not respond to initial silver nitrate chemical cauterization, the treatment was subsequently changed to topical application of common salt granules. These cases were included to evaluate the efficacy of salt granules after failure of silver nitrate cauterization.

#### 2.2.2 Rationale for age limit

The lower age limit was set at 2 weeks to include infants who developed umbilical granuloma in the late neonatal period (i.e., between 28 days and 1 month of age). This period is clinically relevant as umbilical granuloma can develop and require treatment during this time. Although neonates are typically defined as infants from birth to 28 days old, extending the age range to 20 weeks allows for a more comprehensive evaluation of the treatment's efficacy in a broader pediatric population.

#### 2.2.3 Exclusion criteria

Infants with systemic diseases, including immune system disorders and severe medical conditions, as well as infants with a history of severe dermatological conditions or other umbilical complications.

### 2.3 Detailed definition of “severe” medical or dermatological conditions

#### 2.3.1 Severe medical conditions

##### 2.3.1.1 Immune system disorders

Conditions such as severe combined immunodeficiency (SCID), chronic granulomatous disease (CGD), or other primary immunodeficiencies that significantly impair the immune response.

##### 2.3.1.2 Chronic systemic diseases

Conditions such as congenital heart disease, chronic renal failure, or severe metabolic disorders that require ongoing medical management and may affect the healing process or overall health status of the infant.

##### 2.3.1.3 Recent major illness or surgery

Infants who have recently undergone major surgery or have been hospitalized for a severe illness within the past 30 days.

#### 2.3.2 Severe dermatological conditions

##### 2.3.2.1 Extensive skin infections

Infants with a history of extensive skin infections, such as impetigo or cellulitis, that have required systemic antibiotic therapy.

##### 2.3.2.2 Chronic skin conditions

Conditions such as severe eczema, psoriasis, or other chronic dermatological conditions that affect a large area of the skin or have required systemic treatment.

##### 2.3.2.3 Previous umbilical complications

Infants with a history of previous umbilical infections, abscesses, or other significant umbilical complications that may complicate the current treatment or interpretation of the results.

The sample for this study comprised 15 male and 10 female infants, with a mean age of 10.8 (ranging from 2.4 to 20.0) weeks. Among these cases, 7 were identified as wide-based granulomas and 18 as narrow-based granulomas. Initially, 22 cases were treated with topical application of common salt granules. For the 3 cases that did not respond to initial silver nitrate chemical cauterization, the treatment was subsequently changed to topical application of common salt granules.

### 2.4 Sample size calculating

Given that this study was a retrospective analysis, the sample size calculation was based on the effective rate (approximately 90%) and the expected cure rate (100%) of salt granule external application for treating umbilical granuloma as reported in previous literature (10, 11). Using the appropriate statistical formula, with a significance level (α) of 0.05 and a power of the test (1-β) of 0.8, the minimum required sample size was determined to be 20 cases. To account for potential loss to follow-up and case exclusions, a total of 25 children were ultimately included in the study to ensure the robustness and reliability of the results.

The sample size calculation was performed using the following formula for a single proportion (12):

$$n={\left(\frac{Z_{\frac{\alpha }{2}}+Z_{\beta }}{\;p_{1}-p_{0}}\right)}^{2}\times (p_{0}\times (1-p_{0}))$$

Where:

• *n* is the required sample size.

• *Z*_*α*/2_ is the *Z*-value for the desired significance level (1.96 for *α* = 0.05).

• *Z*_*β*_ is the *Z*-value for the desired power (0.84 for power = 0.8).

• *p*_1_ is the expected cure rate (1.00).

• *p*_0_ is the effective rate from previous studies (0.90).

• Using this formula, the minimum required sample size was calculated to be 20 cases. To account for potential loss to follow-up and case exclusions, a total of 25 children were ultimately included in the study.

### 2.5 Treatment protocol

#### 2.5.1 Pre-treatment

Gently cleanse the umbilical area using a cotton ball saturated with normal saline, followed by drying with sterile gauze. This step was performed by a trained nurse in the outpatient clinic setting.

#### 2.5.2 Application of salt granules

Evenly sprinkle a sufficient amount of salt granules over the umbilical fossa to ensure comprehensive coverage of the granulation tissue. This step was also performed by a trained nurse in the outpatient clinic setting.

#### 2.5.3 Fixation of treatment

Secure the treated area with medical tape for a minimum duration of 30 min to facilitate optimal contact between the salt granules and the granulation tissue. This step was performed by a doctor in the outpatient clinic setting to ensure proper fixation and adherence to the protocol.

#### 2.5.4 Follow-up care

After the 30-minute period, disinfect the area using an iodophor-soaked cotton ball to prevent secondary infection. This step was performed by a nurse in the outpatient clinic setting.

#### 2.5.5 Treatment frequency and duration

**Figure 1:**
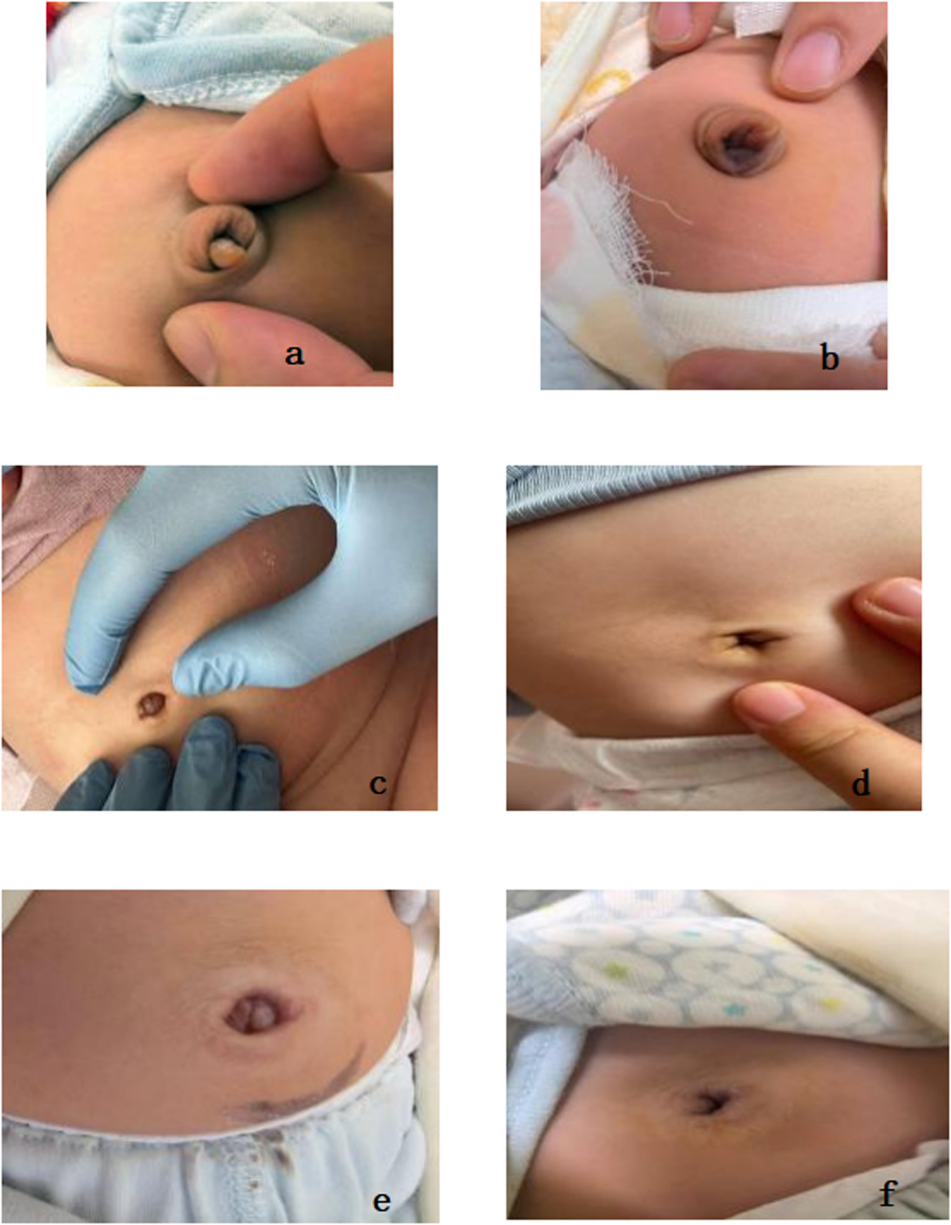
**(a,c,e)** illustrate the appearance of umbilical granuloma prior to treatment. **(b,d,f)** depict the appearance following the detachment of the granuloma after the topical application of common salt granules.

Repeat the aforementioned steps twice daily until complete resolution of the umbilical granuloma is achieved, as evidenced by epithelialization and cessation of exudation. The average treatment duration is 2.5 days (ranging from 1.5 to 9 days), contingent upon the size of the granuloma and the underlying tissue type. (Refer to Figure 1).

### 3.6 Follow-up

During the follow-up period, parents were instructed to continue the treatment at home under the guidance of the healthcare team. The healthcare team provided detailed instructions and demonstrated the treatment procedure to ensure proper application. Parents were also given a contact number to reach out for any questions or concerns during the treatment period.

### 2.7 Primary efficacy endpoint

The primary efficacy endpoint of this study was the complete regression rate of umbilical granuloma, defined as the total disappearance of granulation tissue, satisfactory local epithelialization, and absence of exudation. Secondary efficacy endpoints included treatment duration, complication incidence, and recurrence rate. All pediatric patients were followed up for a period of 3 months to evaluate recurrence.

## 3 Result

All 25 children successfully completed the treatment regimen without any dropouts. This includes the 3 cases that did not respond to initial silver nitrate chemical cauterization and were subsequently treated with topical application of common salt granules. These 3 cases were included in the study to evaluate the efficacy of salt granules after failure of silver nitrate cauterization. The umbilical granulomas in all 25 cases achieved complete regression, resulting in a 100% cure rate. The umbilical granulomas in all cases achieved complete regression, resulting in a 100% cure rate. During the 3-month follow-up period, no recurrence was observed. The average treatment duration was 2.5 days (ranging from 1.5 to 9 days). For smaller, narrow-based or pedunculated umbilical granulomas, the treatment was notably effective within an average of 2 days; conversely, for larger or broad-based lesions, the treatment duration extended to an average of 4–9 days. No treatment-related complications were encountered during the course of therapy. Specifically, there were no instances of skin infection, burns, or other adverse reactions among the participants. The outcomes align with previous research findings and demonstrate a significantly superior therapeutic effect compared to conventional invasive methods such as silver nitrate cauterization and surgical excision. Refer to Table 1 for detailed data.

**Table 1:** Clinical outcomes of the topical application of common salt granules for the treatment of umbilical granuloma in neonates.

Parameters	Wide-based granulomas	Narrow-based granulomas	Total
Sample size (*n*, %)	7 (28%)	18 (72%)	25
Male infant (*n*, %)	5 (71.4%)	10 (55.6%)	15 (60%)
Female infant (*n*, %)	2 (28.6%)	8 (44.4%)	10 (40%)
Average age (weeks)	11.6 (6.8–20.0)	9.6 (2.4–11.2)	10.8 (2.4–20.0)
The minimum duration of treatment (in days)	4	1.5	1.5

## 4 Discussion

Our study demonstrated a 100% cure rate with no recurrence or complications, aligning with recent literature supporting the efficacy of salt granule therapy (10, 11, 13, 14). Notably, the average treatment duration (2.5 days) was shorter than the 5-day regimen reported, likely due to differences in application frequency or granuloma characteristics (15). Importantly, our findings contrast with traditional methods such as silver nitrate cauterization, where complications like chemical burns occurred in 8% of cases (16), underscoring the safety advantage of salt granules. The absence of adverse effects in our cohort further supports its suitability for broad-based and deep-seated lesions, which pose challenges for ligation or electrocautery.

While the etiology of umbilical granulomas remains debated, our results suggest that osmotic dehydration—central to salt granule therapy—effectively addresses inflammatory granulation tissue regardless of underlying causes. This mechanism may explain the rapid resolution observed in narrow-based lesions (average 2 days) compared to broader ones (4–9 days). Future studies should explore correlations between granuloma morphology, etiology, and treatment response.

In light of the underlying inflammatory response mechanism, initial attempts by some researchers to use topical antibiotics such as mupirocin yielded unsatisfactory results. This suggests that granuloma formation may not be exclusively attributed to bacterial infection, and antibiotic therapy alone is insufficient to address the issue. Several studies have reported promising outcomes with the use of steroids in treating umbilical granulomas. However, concerns arise regarding the prolonged application (3–4 weeks) of high-potency steroids on highly vascularized granuloma tissue, which may result in systemic absorption and suppression of the hypothalamic-pituitary-adrenal (HPA) axis. Additionally, there is an increased risk of local adverse effects such as infection, skin hypopigmentation, and atrophy, thereby limiting its widespread adoption and clinical application (17, 18).

Currently, there is no consensus regarding the optimal treatment for umbilical granuloma. Most standard reference texts recommend the use of 75% silver nitrate cauterization to treat granulation tissue, with the procedure repeated every few days until the base is fully desiccated. While this method has proven efficacy, complications such as chemical burns to the surrounding skin have been reported in the literature (8). In our study, three pediatric patients experienced treatment failure following initial silver nitrate cauterization, with two cases developing burns around the umbilical ring. The likely reasons for these adverse events include the relatively large size and deep location of the granulomas within the umbilical depression, which necessitated multiple cauterizations and posed challenges for adequate exposure, thereby prolonging the treatment course and increasing the risk of skin injury. Similarly, our institution has extensively utilized electrocautery for the treatment of umbilical granuloma. However, for lesions situated in deeper locations, adequate exposure poses a significant challenge. Additionally, due to the crying and restlessness commonly observed in pediatric patients, their compliance with the procedure is suboptimal, leading to frequent reports of superficial burns to the surrounding skin (19).

Furthermore, considering that granulation tissue lacks nerve innervation and consequently does not elicit pain sensation, certain scholars have proposed the use of silk thread ligation for the treatment of umbilical granulomas. However, in clinical practice, this method is most appropriate for granulomas that are prominently protruding, with a narrow base and pedunculated morphology. For deep-seated, broad-based, or sessile granuloma lesions, the procedure becomes considerably more challenging, and the therapeutic outcomes are less satisfactory. In cases where non-invasive or minimally invasive treatments prove ineffective, many medical institutions opt to perform granuloma excision under general anesthesia. Nevertheless, this invasive approach is often deemed unacceptable by the parents of pediatric patients.

The use of sodium chloride for the treatment of umbilical granuloma is based on the principle of desiccation: the creation of a local hypertonic environment facilitates osmotic water absorption from cells, leading to dehydration, contraction, and eventual necrosis of the moist granulation tissue (20). Human skin, being well-keratinized, serves as an effective protective barrier against osmotic reactions between sodium chloride and the periumbilical skin, ensuring that this targeted therapy does not compromise the integrity of surrounding tissues. In this study, the shortest treatment duration was 1.5 days, while the longest was 9 days. Our findings indicate a significant correlation between treatment duration and the size as well as the base characteristics of the granulomas. Specifically, granulomas that were smaller in size, had a narrower base, or were pedunculated exhibited a markedly shortened onset time and were more prone to detachment. A randomized controlled trial conducted in Malaysia in 2022 compared the efficacy of salt and copper sulfate (CuSO4) in treating umbilical granuloma, revealing a significantly higher complete regression rate in the salt group compared to the CuSO4 group (15). A recent systematic review indicated that the effectiveness of salt treatment for umbilical granuloma exceeded 90% (21). This study demonstrated that the application of salt granules externally resulted in a 100% cure rate for umbilical granuloma, with no instances of recurrence or complications. The most recent systematic review, graded as Level I evidence, encompassed 24 pertinent studies conducted globally and concluded that the external application of salt granules for the treatment of umbilical granuloma is a straightforward, efficacious, and cost-effective method that does not result in recurrence or complications (20).

It is important to highlight that prior literature has documented the efficacy of high-concentration saline solutions in addressing umbilical granuloma issues. For instance, Daruwalla et al.'s study (22) demonstrated that high-concentration saline solution dehydrates granulation tissue via localized osmotic pressure, thereby achieving therapeutic outcomes. In this investigation, however, salt granules were applied directly to the affected area, with a therapeutic mechanism analogous to that of high-concentration saline solutions. Notably, the hypertonic environment created by the salt granules is more pronounced, leading to a more rapid dehydration and contraction of the granulation tissue. Further research is warranted to compare the benefits and drawbacks of these two approaches in order to determine which method is more suitable for infants.

This study has several limitations. First, the sample size, though statistically calculated, remains modest, which may limit the generalizability of the results. Second, the retrospective design and absence of a control group preclude direct comparisons with alternative treatments. Future research should prioritize large-scale, prospective RCTs to confirm these findings and assess long-term outcomes.

This single-center, retrospective study had a limited sample size of 25 cases. Although some phase I clinical trials consider a sample size of 20–80 to be sufficient for initial efficacy assessments, our sample size is at the lower end of this range. This limitation restricts the robustness of our inferential conclusions and the generalizability of our findings. The study design is more akin to an observational study from medical records on the successful salt treatment, rather than a prospective interventional clinical analysis. Future research should incorporate a control group and conduct large-scale, multi-center, prospective studies to further validate the efficacy of topical salt granule application. Additionally, high-quality randomized controlled trials (RCTs) or cohort studies are necessary to substantiate the findings and establish the treatment as a first-line option.

## 5 Conclusion

Topical application of common salt granules appears to be a simple, effective, safe, and cost-effective treatment for infant umbilical granuloma, based on our retrospective analysis. However, the lack of a control group and the retrospective nature of the study mean that our conclusions should be interpreted with caution. Further prospective studies with control groups are needed to confirm these findings and establish the treatment as a first-line option.

## Data Availability

The original contributions presented in the study are included in the article/Supplementary Material, further inquiries can be directed to the corresponding author/s.
